# Supplementary material for: Polyketide synthase gene clusters in the ericoid mycorrhizal fungus Oidiodendron maius: genome-wide prediction, expression profiling, and functional characterization of knockout mutants
Source: Front Plant Sci. 2026 Sep 16;17:1924430. doi: 10.3389/fpls.2026.1924430 (PMC13623903; doi:10.3389/fpls.2026.1924430)
Supplement: Supplementary file 1 [file SupplementaryFile1.zip › Supplementary Materials/Supplementary Folder 1.zip!/Oidma1_AssemblyScaffolds_Repeatmasked/knownclusterblast/cluster54/jgi.p_Oidma1_138671_mibig_hits.html]

| MIBiG Protein | Description | MIBiG Cluster | MiBiG Product | % ID | % Coverage | BLAST Score | E-value |
| --- | --- | --- | --- | --- | --- | --- | --- |
| BAD97695.1 | Aft10-1 | BGC0000003 | AF-toxin\_biosynthetic\_gene\_cluster | 48 | 97.2789115646 | 401 | 1.3e-111 |
| EWG54268.1 | hypothetical\_protein | BGC0001190 | Fusaric\_acid\_biosynthetic\_gene\_cluster | 42 | 93.8775510204 | 329 | 6.1e-90 |
| CBF80495.1 | Acyl-CoA\_dehydrogenase\_(Eurofung) | BGC0000959 | Aspyridone\_biosynthetic\_gene\_cluster | 37 | 97.5056689342 | 277 | 2.7e-74 |
| ABO15870.1 | acyl-CoA\_dehydrogenase | BGC0000130 | Pyrrolomycin\_biosynthetic\_gene\_cluster | 29 | 88.4353741497 | 159 | 8.3e-39 |
| ACO31278.1 | PtmO4 | BGC0001140 | Platensimycin\_/\_platencin\_biosynthetic\_gene\_cluster | 30 | 87.7551020408 | 150 | 3.9e-36 |
| AJI44179.1 | hypothetical\_protein | BGC0001193 | Thiolutin\_biosynthetic\_gene\_cluster | 28 | 87.9818594104 | 144 | 3.6e-34 |
| AFY23017.1 | isovaleryl-CoA\_dehydrogenase | BGC0000274 | Tetarimycin\_biosynthetic\_gene\_cluster | 30 | 90.022675737 | 142 | 1.1e-33 |
| EFG10341.1 | Acyl-CoA\_dehydrogenase\_domain\_protein | BGC0000373 | Holomycin\_biosynthetic\_gene\_cluster | 29 | 89.1156462585 | 141 | 2.3e-33 |
| ADZ24990.1 | prolyl-ACP\_dehydrogenase | BGC0000380 | Leupyrrin\_biosynthetic\_gene\_cluster | 32 | 67.5736961451 | 119 | 7.3e-27 |
| ABB69751.1 | PlaP3 | BGC0000654 | Phenalinolactone\_biosynthetic\_gene\_cluster | 28 | 61.9047619048 | 95 | 1.5e-19 |
| ESP90846.1 | acyl-CoA\_dehydrogenase | BGC0000891 | Bromophenols\_/\_bromopyrroles\_biosynthetic\_gene\_cluster | 26 | 67.5736961451 | 91 | 3.6e-18 |
| AAG29788.2 | acyl-CoA\_dehydrogenase | BGC0000833 | Coumermycin\_A1\_biosynthetic\_gene\_cluster | 25 | 64.8526077098 | 85 | 1.5e-16 |
| AHA34036.1 | Bmp3 | BGC0000890 | Bromophenols\_/\_bromopyrroles\_biosynthetic\_gene\_cluster | 28 | 51.0204081633 | 84 | 2.6e-16 |
| AAD24879.1 | putative\_acyl-CoA\_dehydrogenase | BGC0000127 | Pyoluteorin\_biosynthetic\_gene\_cluster | 30 | 50.7936507937 | 78 | 2.4e-14 |
| ABY83172.1 | Azi34 | BGC0000960 | Azinomycin\_B\_biosynthetic\_gene\_cluster | 30 | 36.2811791383 | 74 | 4.6e-13 |
